# Integrated morbidity management for lymphatic filariasis and podoconiosis, Ethiopia

**DOI:** 10.2471/BLT.16.189399

**Published:** 2017-06-26

**Authors:** Kebede Deribe, Biruck Kebede, Mossie Tamiru, Belete Mengistu, Fikreab Kebede, Sarah Martindale, Heven Sime, Abate Mulugeta, Biruk Kebede, Mesfin Sileshi, Asrat Mengiste, Scott McPherson, Amha Fentaye

**Affiliations:** a Wellcome Trust Brighton and Sussex Centre for Global Health Research, Brighton and Sussex Medical School, Falmer, Brighton, East Sussex, BN1 9PX, England.; b NTD case team, Diseases Prevention and Control Directorate, Federal Ministry of Health, Addis Ababa, Ethiopia.; c RTI International, Addis Ababa, Ethiopia.; d Center for Neglected Tropical Diseases, Liverpool School of Tropical Medicine, Liverpool, England.; e Ethiopian Public Health Institute, Addis Ababa, Ethiopia.; f Country Office, World Health Organization, Addis Ababa, Ethiopia.; g National Podoconiosis Action Network, Addis Ababa, Ethiopia.

## Abstract

**Problem:**

Lymphatic filariasis and podoconiosis are the major causes of tropical lymphoedema in Ethiopia. The diseases require a similar provision of care, but until recently the Ethiopian health system did not integrate the morbidity management.

**Approach:**

To establish health-care services for integrated lymphoedema morbidity management, the health ministry and partners used existing governmental structures. Integrated disease mapping was done in 659 out of the 817 districts, to identify endemic districts. To inform resource allocation, trained health extension workers carried out integrated disease burden assessments in 56 districts with a high clinical burden. To ensure standard provision of care, the health ministry developed an integrated lymphatic filariasis and podoconiosis morbidity management guideline, containing a treatment algorithm and a defined package of care. Experienced professionals on lymphoedema management trained government-employed health workers on integrated morbidity management. To monitor the integration, an indicator on the number of lymphoedema-treated patients was included in the national health management information system.

**Local setting:**

In 2014, only 24% (87) of the 363 health facilities surveyed provided lymphatic filariasis services, while 12% (44) provided podoconiosis services.

**Relevant changes:**

To date, 542 health workers from 53 health centres in 24 districts have been trained on integrated morbidity management. Between July 2013 and June 2016, the national health management information system has recorded 46 487 treated patients from 189 districts.

**Lessons learnt:**

In Ethiopia, an integrated approach for lymphatic filariasis and podoconiosis morbidity management was feasible. The processes used could be applicable in other settings where these diseases are co-endemic.

## Résumé

**Problème:**

La filariose lymphatique et la podoconiose sont les principales causes du lymphœdème tropical en Éthiopie. Ces maladies requièrent une prestation de soins similaire, mais en 2012, le système de santé éthiopien n'a pas intégré la gestion de la morbidité.

**Approche:**

Le ministère de la Santé et ses partenaires ont utilisé les structures gouvernementales existantes pour mettre en place des services de santé en vue de la gestion intégrée de la morbidité liée au lymphœdème. Une cartographie intégrée de la maladie a été réalisée dans 659 des 817 districts pour repérer ceux où elle était endémique. Afin d'orienter l'affectation des ressources, des agents de vulgarisation sanitaire qualifiés ont intégré des évaluations de la charge de morbidité dans 56 districts présentant une charge clinique élevée. Pour assurer une prestation standard de soins, le ministère de la Santé a rédigé des directives sur la gestion intégrée de la morbidité liée à la filariose lymphatique et à la podoconiose. Ces directives comportent un algorithme de traitement et un programme de soins précis. Des professionnels possédant de l'expérience dans la gestion du lymphœdème ont formé des agents de santé employés par le gouvernement à la gestion intégrée de la morbidité. Pour suivre l'intégration, un indicateur relatif au nombre de patients pris en charge pour un lymphœdème a été inclus dans le système national d'information sanitaire.

**Environnement local:**

En 2014, seuls 24% (87) des 363 établissements de santé participant à l'enquête ont fourni des services liés à la filariose lymphatique et 12% (44) ont fourni des services liés à la podoconiose.

**Changements significatifs:**

À ce jour, 542 agents de santé provenant de 53 centres de santé répartis dans 24 districts ont été formés à la gestion intégrée de la morbidité. Entre juillet 2013 et juin 2016, le système national d'information sanitaire a enregistré 46 487 patients pris en charge dans 189 districts.

**Leçons tirées:**

La mise en place d'une approche intégrée pour la gestion de la morbidité liée à la filariose lymphatique et à la podoconiose a été possible en Éthiopie. Les processus utilisés pourraient être appliqués dans d'autres environnements où ces maladies sont co-endémiques.

## Resumen

**Situación:**

La filariasis linfática y la podoconiosis son las mayores causas del linfedema tropical en Etiopía. Las enfermedades requieren una atención sanitaria similar, pero en 2012 el sistema sanitario de Etiopía no integró la gestión de la morbilidad.

**Enfoque:**

Para establecer servicios sanitarios para la gestión integrada de la morbilidad por linfedema, el ministerio de salud y colaboradores utilizaron estructuras gubernamentales existentes. Se realizó un mapeo integrado de la enfermedad en 659 de 817 distritos para identificar los distritos endémicos. Para informar sobre la asignación de recursos, agentes formados de extensión sanitaria integraron evaluaciones sobre la carga de la enfermedad en 56 distritos con una carga clínica elevada. Para garantizar un suministro estándar de la atención, el ministerio de salud desarrolló unas directrices para la gestión integrada de la morbilidad de la filariasis linfática y la podoconiosis, las cuales contenían un algoritmo de tratamiento y un paquete definido de cuidados. Profesionales con experiencia en la gestión del linfedema formaron a trabajadores de la salud empleados por el gobierno sobre la gestión integrada de la morbilidad. Para controlar la integración, se incluyó un indicador del número de pacientes tratados de linfedema en el sistema nacional de información para la gestión de la salud.

**Marco regional:**

En 2014, solo el 24% (87) de los 363 centros sanitarios encuestados ofrecían servicios para la filariasis linfática, mientras que el 12% (44) ofrecían servicios para la podoconiosis.

**Cambios importantes:**

Hasta la fecha, 542 trabajadores sanitarios de 53 centros de salud en 24 distritos han sido formados acerca de la gestión integrada de la morbilidad. Entre julio de 2013 y junio de 2016, el sistema nacional de información para la gestión de la salud registró 46 487 pacientes tratados de 189 distritos.

**Lecciones aprendidas:**

En Etiopía, fue viable un enfoque integrado de la gestión de la morbilidad para la filariasis linfática y la podoconiosis. Los procesos utilizados podrían ser aplicables en otros lugares donde estas enfermedades son coendémicas.

## ملخص

**المشكلة:**

يعتبر داء الفيلاريات اللمفي وداء الفيل ****هما السببان الرئيسيان للإصابة بمرض الوذمة اللمفية الاستوائي في إثيوبيا. ويتطلب هذان المرضان توفير رعاية مماثلة، إلا أنه في عام 2012 لم يقم النظام الصحي الإثيوبي بإدراج الإدارة المختصة بمعدلات الإصابة بالمرض.

**الأسلوب:**

لإنشاء خدمات الرعاية الصحية للإدارة المتكاملة لمعدلات الإصابة بالوذمة اللمفية، قامت وزارة الصحة، بالتعاون مع شركائها، باستخدام الهياكل الحكومية القائمة. ولتحديد المناطق الموبوءة، تمت الاستعانة بالترسيم المتكامل للأمراض، وذلك في 659 منطقة من أصل 817 منطقة موبوءة. وللاطلاع على توزيع الموارد، قام العاملون المدربون في مجال الإرشاد الصحي بتقييمات متكاملة لعبء المرض في 56 منطقة لديها عبء سريري مرتفع. ولضمان توفير رعاية معيارية، قامت وزارة الصحة بتطوير الإرشادات الإدارية لمعدلات الإصابة بداء الفيلاريات اللمفي وداء الفيل بما يشتمل على خوارزمية للعلاج ومجموعة محددة للرعاية. ويقوم مختصون ذوو خبرة في إدارة مرض الوذمة اللمفية بتدريب العاملين الحكوميين في مجال الصحة، على الإدارة المتكاملة لمعدلات الإصابة المرض. ولمراقبة التكامل فإنه تم وضع مؤشر لعدد المرضى الذين تم علاجهم من مرض الوذمة اللمفية ضمن نظام المعلومات الوطني للإدارة الصحية.

**المواقع المحلية:**

في عام 2014 قامت ‏24% (87) من أصل 363 منشأة صحية بإجراء مسح للخدمات المقدمة لمرضى داء الفيلاريات اللمفي، في حين قامت ‏12% (44) منشأة بتقديم خدمات لمرضي داء الفيل.

**التغييرات ذات الصلة:**

حتى الآن تم تدريب 542 من العاملين في مجال الصحة من 53 مركزاً صحياً في 24 منطقة، على الإدارة المتكاملة لمعدلات الإصابة بالمرض. وفي الفترة ما بين يوليو/تموز 2013 ويونيو/حزيران 2016، سجل نظام المعلومات الوطني للإدارة الصحية 46487 مريضاً تم علاجهم من 189 منطقة.

**الدروس المستفادة:**

ثبتت جدوى استخدام النهج المتكامل لمعدلات الإصابة بداء الفيلاريات اللمفي وداء الفيل في إثيوبيا. ويمكن تطبيق العمليات المستخدمة في مواقع أخرى تستوطن فيها هذه الأمراض.

## 摘要

**问题:**

淋巴丝虫病和象皮肿是造成埃塞俄比亚热带淋巴水肿的主要原因。 这些疾病需要提供类似的护理，但是在 2012 年，埃塞俄比亚卫生系统没有整合发病率管理。

**方法:**

为了建立用于综合发病率管理的医疗护理服务，卫生部和合作伙伴使用了现有的政府体系。 817 个地区中，659 个地区绘制了综合疾病地图，用以确定疾病流行地区。 为提供资源分配信息，经过培训的健康推广工作人员在 56 个临床负担高的地区开展了综合疾病负担评估。 为了确保提供标准护理，卫生部制定了综合淋巴丝虫病和象皮肿发病率管理指南，包含治疗算法和既定的护理包。 在淋巴水肿控制方面经验丰富的专家就综合发病率管理对政府雇佣的卫生工作人员进行了培训。 为了监控综合管理，国家卫生管理信息系统中纳入了一个显示接受淋巴水肿治疗的患者人数的指标。

**当地状况:**

2014 年，被调查的 363 个卫生机构中，仅 24% (87) 个提供淋巴丝虫病护理服务，12% (44) 个提供象皮肿护理服务。

**相关变化:**

截至目前，24 个地区 53 家卫生中心的 542 名卫生工作人员接受了综合发病率管理培训。 在 2013 年 7 月至 2016 年 6 月期间，国家卫生管理信息系统记录了来自 189 个地区的 46487 名接受治疗的患者。

**经验教训:**

在埃塞俄比亚，对淋巴丝虫病和象皮肿进行综合发病率管理的方法是可行的。 这些流程也可能适用于这类疾病并发流行的其他地区。

## Резюме

**Проблема:**

Лимфатический филяриатоз и подокониоз являются основными причинами тропической лимфедемы в Эфиопии. Заболевания требуют аналогичного лечения, но в 2012 году система здравоохранения Эфиопии не использовала комплексный подход к управлению заболеваемостью.

**Подход:**

С целью создания медицинских услуг для комплексного регулирования заболеваемости лимфедемой Министерство здравоохранения и партнеры использовали существующие правительственные структуры. Для выявления эндемичных районов было проведено комплексное картографирование болезней в 659 из 817 районов. Чтобы информировать о распределении ресурсов, подготовленные работники здравоохранения провели комплексные оценки бремени болезни в 56 районах с высокой клинической нагрузкой. Чтобы обеспечить оказание стандартной медицинской помощи, Министерство здравоохранения разработало руководство по комплексному управлению заболеваемостью лимфатическим филяриатозом и подокониозом, содержащее алгоритм лечения и определенный пакет медицинской помощи. Опытные специалисты по лечению лимфедемы обучили государственных работников здравоохранения комплексному управлению заболеваемостью. Для мониторинга интеграции в национальную информационную систему управления здравоохранением был включен показатель количества пациентов, получавших лечение при лимфедеме.

**Местные условия:**

В 2014 году только 24% (87) из 363 обследованных медицинских учреждений оказывали медицинскую помощь при лимфатическом филяриатозе и 12% (44) медицинских учреждений оказывали медицинскую помощь при подокониозе.

**Осуществленные перемены:**

На сегодняшний день 542 медицинских работника из 53 медицинских центров в 24 районах прошли подготовку по вопросам комплексного управления заболеваемостью. В период с июля 2013 года по июнь 2016 года в национальной информационной системе управления здравоохранением было зарегистрировано 46 487 получавших лечение пациентов из 189 районов.

**Выводы:**

В Эфиопии был внедрен комплексный подход к регулированию заболеваемости лимфатическим филяриатозом и подокониозом. Используемые процессы могут применяться в других условиях, для которых эти заболевания являются эндемичными.

## Introduction

Lymphatic filariasis and podoconiosis are major causes of lymphoedema in tropical areas.^1^ Lymphatic filariasis is a mosquito-borne parasitic infection, while podoconiosis is an inflammatory disease caused by prolonged contact with irritant soil minerals. However, both diseases require a similar provision of health care.

People with lymphoedema caused by lymphatic filariasis need access to care throughout their lives^2^ and the World Health Organization (WHO) has suggested a minimum package of care for managing morbidity and preventing disability. The package includes: providing antifilarial medicine, either through mass drug administration or individual treatment; hydrocele surgery; preventing and treating episodes of adenolymphangitis; and managing the lymphoedema.^3^

Podoconiosis causes lymphoedema of the lower limb and acute pain.^4^ Early stages of the disease are reversible, but more advanced stages need lifelong treatment. The main prevention methods are use of footwear, regular foot hygiene and floor coverings, whereas already affected people receive management of their lymphoedema-related morbidity. The management includes daily foot hygiene using soap, water and antiseptics, emollients to restore skin function, elevation of the legs, exercise, use of socks and shoes, and if needed bandaging and removal of nodules.^4^

Following the Global Programme to Eliminate Lymphatic Filariasis – which aims to eliminate lymphatic filariasis as a public health problem by 2020^5^^,^^6^ – the *Second edition of national neglected tropical diseases master plan* of Ethiopia for 2016–2020 targets lymphatic filariasis and podoconiosis for elimination by 2020 and 2030, respectively.^7^ The health ministry has taken an integrated approach for care provision, because it was not feasible to differentiate between the two diseases at primary health-care facilities and because of the similarity in health-care services provided.

Here we describe the implementation of the integrated approach into the Ethiopian health system.

## Local setting

In Ethiopia, lymphatic filariasis is endemic in 70 districts, with over 5.6 million people at risk of acquiring the disease;^7^ whereas podoconiosis is endemic in 345 districts, with 34.9 million people at risk.^7^^,^^8^ Twenty-nine of these districts are co-endemic.

In 2014, 24% (87) of the 363 health facilities surveyed provided lymphatic filariasis services, while 12% (44) provided podoconiosis services.^9^ In the endemic districts, 42 nongovernmental partner-supported centres provide treatment for lymphatic filariasis and podoconiosis. These treatment centres have experienced staff members and act as training centres for health workers on lymphoedema morbidity management.

## Approach

### Mapping and burden assessment

In 2013, the mapping teams for the two diseases joined together to co-map the disease distribution in 659 of Ethiopia’s 817 districts. Details on the integrated mapping are described elsewhere.^10^

To estimate the allocation of resources needed for morbidity management and disability prevention, implementing partners carried out a burden assessment to know the exact number of people with lymphoedema and/or hydrocele in the endemic districts. In 2015, an integrated burden assessment pilot was carried out in 20 co-endemic districts to inform the development of a national burden assessment protocol at district-level in 2016. The protocol describes the standardized design and implementation procedure.

Before the assessment started, health professionals from the partner-supported treatment centres provided a one-day classroom training course for health extension workers at a central place in each district. The training entailed: how to identify people with lymphoedema and hydrocele by using pictures on different stages of each disease, signs and symptoms; how to treat and prevent lymphoedema; and how to use the patient’s questionnaire for the burden assessment. The participants received travel reimbursement.

The health extension workers informed the health development teams about the assessment, and they passed the information to the community by visiting each household. Subsequently, the health extension workers visited each household to identify people with lymphoedema or hydroceles. They provided information on morbidity management, self-care and where to seek care. If nobody was at home at the time of the visit, they left a message with neighbours that they would return by the end of the day or the next morning. They followed up twice and if nobody could be reached, they reported the household as absent. To assess if lymphoedema was correctly identified, health extension workers were instructed in selected districts to refer identified people for verification by health officers or nurses at central locations, usually health centres, on a specified day.

Between 2015 and 2016 the burden assessment identified 44 039 lymphoedema and 1574 hydrocele cases in 56 districts. Twenty five of these districts had cases of both lymphoedema and hydrocele.

### Joint technical working group

The health ministry organizes technical working groups to provide evidence-based technical and implementation inputs to health programmes. The groups, which meet monthly, include health ministry staff, members of research institutes, implementing partners, international organizations and donors. To aid the implementation of the integrated approach and enable a transparent discussion, the ministry combined the lymphatic filariasis and podoconiosis technical working groups. The ministry also assigned a focal person for the two diseases, whose role is to plan, coordinate and oversee the implementation of the interventions. In addition, this person is responsible for organizing and leading the technical working group meetings.

### Guideline development

To develop an integrated morbidity management and disability prevention guideline, which would facilitate streamlining the integrated morbidly management into the general health system, the health ministry hosted workshops with the joint technical working group in 2015.^11^ To identify the minimum health service package, the group, with support from experts in the field, reviewed national and global experiences on morbidity management.^3^ The new guideline contains a simple algorithm on clinical assessment, treatment and referral needs, and a defined care package.^11^ The package includes patient counselling and teaching of a self-care routine, foot hygiene, skin care with ointment or emollients, leg elevation and exercise, footwear, wound care and management of adenolymphangitis and, if needed, bandaging for people with podoconiosis. Based on the severity of disease, health workers encourage newly diagnosed patients either to report to the clinic or be visited by a health worker in their homes once a month for the first three months. During follow-up visits, health workers monitor the lymphoedema progress, look for entry lesions, remind patients and their families of the defined package and the importance of prevention and early care, and provide patients with more treatment supplies. After the initial three months, patients are followed up annually to address any issues related to morbidity management and to ensure compliance with the self-care routine.

### Implementation

In the districts or nearby towns in adjacent districts, experienced professionals on lymphoedema management provide a three-day guideline course for government-employed health workers. The first two days contain lectures on neglected tropical diseases in general, details on the two diseases and morbidity management. On day three, the participants receive practical training on morbidity management. So far, 542 workers from 53 health centres in 24 districts have been trained. Based on supportive supervision reports performed by partners and health ministry staff, health workers are providing services according to the national guideline.

The health ministry, supported by partners, also developed and rolled out a teaching video for health workers on integrated morbidity management.

The implementation also requires some additional resources, such as pamphlets on the self-care routine, treatment supplies and custom-made shoes. Hence, the morbidity management services and training on self-care are being scaled up in a phased approach at health centres in the endemic districts.

### Monitoring and evaluation

Since July 2013, the national health management information system has contained an indicator for the number of lymphoedema-treated patients, segregated by cause (if available), which enables monitoring and evaluation of the integrated approach. The indicator definition of lymphoedema is a chronic progressive swelling of one or more parts of the body due to accumulation of lymphatic fluid and the fluid is gradually replaced by fibrous tissue. Treated patients are those who have received training on self-care routines and returned for the three-month follow-up. Health workers record demographic information, including name, contact address, sex, age, age of onset of condition, clinical stage and presence of wounds/entry lesions for new patients. An information system focal person collects reports on the number of lymphoedema cases treated from the registers in each health centre and manually enters the information into the health management information system. The information is then sent to the district health office where the reports are compiled and sent to the regional health bureaus. Treated patients are only reported once and between July 2013 and June 2016 the information system had recorded 46 487 treated patients from 189 districts.

## Lessons learnt

The implementation of integrated morbidity management for lymphatic filariasis and podoconiosis has worked well. However, some organizations and budgets focused only on one of the diseases, which limited the full implementation at regional, zonal and districts levels.

Several factors contributed to the successful implementation. First, the health helped to convert the vertical programmes to an integrated programme. Second, the presence of health professionals experienced in lymphoedema management supported the implementation through training of health workers, though these experts were not available in all endemic districts. Third, the existing treatment centres served as practical demonstration sites. Finally, committed partners supported the implementation of the integrated approach technically and financially (Box 1).

Box 1 **Summary of main lessons learnt**

- An effective health ministry leadership helped the implementation efforts through the development of the national guideline for the integrated morbidity management and disability prevention.
- The presence of treatment centres and experienced health workers on lymphoedema morbidity management within the country was important for training of health workers.
- The involvement of committed partners from planning stage to implementation contributed to the successful integration.

The integrated approach during the mapping and burden assessments reduced cost in comparison to the disease-specific approach. According to the planning budgets covering 659 districts, the estimated cost of lymphatic filariasis mapping was 1 212 209 United States dollars (US$), while the budget for podoconiosis mapping was estimated at US$ 1 211 664, compared to the actual cost of the dual mapping of US$ 1 291 400. Team training, one diagnostic test for both diseases, supplies and travel contributed to most of the savings.^10^ By integrating the two diseases in the burden assessment, the need for a diagnostic disease-specific test was unnecessary, which reduced staff time and cost. Furthermore, having a single indicator has eased advocacy for the inclusion of the indicator into the information system, leading to regular and sustainable data collection. Finally, the development of a guideline brought partners and experts together to discuss experiences and resolve implementation differences, such as the use of bandaging and surgical removal of nodules for podoconiosis cases.^11^ The experts agreed that most aspects of lymphoedema management can be integrated, while maintaining disease specific parts. This process helped to capitalize on national experience while also learning from global experiences.^3^

The lessons learnt in Ethiopia could be used by other co-endemic countries, such as Brazil, India and the United Republic of Tanzania, wishing to implement an integrated morbidity management approach. In the future, the approach could include other neglected tropical diseases causing similar morbidities, such as leprosy and Buruli ulcer.^12^
